# Brain Derived Neurotrophic Factor (BDNF) Delays Onset of Pathogenesis in Transgenic Mouse Model of Spinocerebellar Ataxia Type 1 (SCA1)

**DOI:** 10.3389/fncel.2018.00509

**Published:** 2019-01-21

**Authors:** Aaron Mellesmoen, Carrie Sheeler, Austin Ferro, Orion Rainwater, Marija Cvetanovic

**Affiliations:** ^1^Department of Neuroscience, University of Minnesota, Minneapolis, MN, United States; ^2^Department of Lab Medicine and Pathology, University of Minnesota, Minneapolis, MN, United States; ^3^Institute for Translational Neuroscience, University of Minnesota, Minneapolis, MN, United States

**Keywords:** ATAXIN-1, BDNF, astroglia, neuroprotective, nuclear factor κb, cerebellum, neurodegeneration

## Abstract

Spinocerebellar ataxia type 1 (SCA1) is a fatal neurodegenerative disease caused by an abnormal expansion of CAG repeats in the *Ataxin-1 (ATXN1)* gene and characterized by motor deficits and cerebellar neurodegeneration. Even though mutant ATXN1 is expressed from an early age, disease onset usually occurs in patient’s mid-thirties, indicating the presence of compensatory factors that limit the toxic effects of mutant ATXN1 early in disease. Brain derived neurotrophic factor (BDNF) is a growth factor known to be important for the survival and function of cerebellar neurons. Using gene expression analysis, we observed altered BDNF expression in the cerebella of Purkinje neuron specific transgenic mouse model of SCA1, *ATXN1[82Q]* mice, with increased expression during the early stage and decreased expression in the late stage of disease. We therefore investigated the potentially protective role of BDNF in early stage SCA1 through intraventricular delivery of BDNF *via* ALZET osmotic pumps. Extrinsic BDNF delivery delayed onset of motor deficits and Purkinje neuron pathology in *ATXN1[82Q]* mice supporting its use as a novel therapeutic for SCA1.

## Introduction

Spinocerebellar ataxia type 1 (SCA1) is a dominantly inherited and fatal neurodegenerative disease resulting from an overexpansion of CAG repeats within the *Ataxin-1 (ATXN1)* gene (Banfi et al., 1996; Zoghbi and Orr, 2009). SCA1 belongs to a group of polyglutamine (polyQ) disorders that also includes SCA2, 3, 6, 7, 17, spinobulbar muscular atrophy, Huntington’s disease (HD), and dentatorubropallidoluysian atrophy (Genis et al., 1995; Gusella and MacDonald, 2000; La Spada and Taylor, 2010). Clinical onset of SCA1 is characterized by ataxia, or loss of motor coordination and balance, which typically presents during patient’s mid-thirties (Genis et al., 1995; Orr and Zoghbi, 2007; Rüb et al., 2013; Matilla-Dueñas et al., 2014). Progressive degradation of motor function leads to death within 10–20 years following clinical onset (Rüb et al., 2013). Currently, no treatments exist for SCA1 (Paulson et al., 2017).

**Figure 1:**
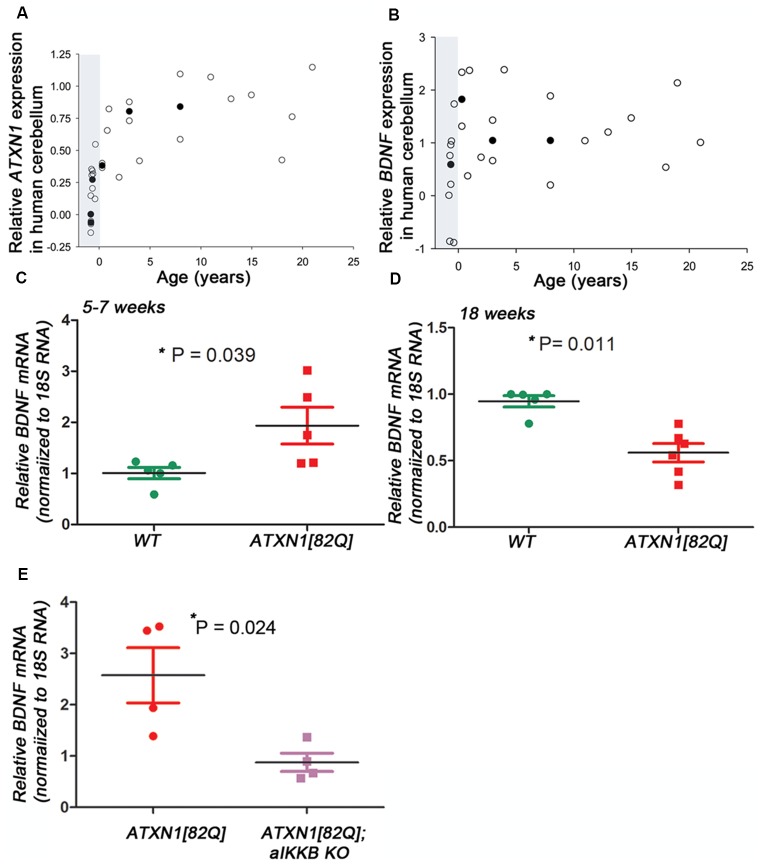
Brain derived neurotrophic factor (BDNF) expression in transgenic spinocerebellar ataxia type 1 (SCA1) mice. **(A–B)** Relative *Ataxin-1* (ATXN1) **(A)** and BDNF **(B)** expression in the human cerebellar cortex [polyglutamine (polyQ) sequence/SCA1 status unknown] obtained from the transcriptome data at *Allen Brain Institute*. Raw reads per kilobase million (RPKM) values were normalized to the highest and lowest values and data was averaged when multiple patient samples fell within the same postnatal year or same post conception week. Open circles represent single patient samples and closed circles represent average expression (used when possible). Shaded areas mark pre-natal period. **(C–E)** Reverse transcription and quantitative polymerase chain reaction (RT-qPCR) using cerebellar extracts from **(C)** early stage (5–7 weeks) and **(D)** late stage (18–24 weeks old) *ATXN1[82Q]* mice. Results are normalized using 18S RNA and age-matched wild-type (WT) littermates. **(E)** RT-qPCR using cerebellar extracts from *ATXN1[82Q]* mice and mice in which astroglialnuclear factor κ-light-chain-enhancer of activated B cells (NF-κB) is inhibited early indisease [*ATXN1[82Q]*; aIKKβ KO]. Results are normalized using 18S RNA and age-matched WT littermates. For **(C–E)** error bars = SEM. Student’s *t*-test *P* values. Each dot represents a biological sample.

ATXN1 is expressed prior to birth in humans [Figure 1A, created using human developmental transcriptome data from BrainSpan (Miller et al., 2014)], yet the clinical phenotype of SCA1 emerges decades later. Similarly, in *ATXN1[82Q]* mice, Purkinje neuron specific transgenic mouse model of SCA1, mutant ATXN1 is expressed from postnatal day 10 (P10; Serra et al., 2006) yet they exhibit motor deficits and Purkinje neuron pathology earliest at 12 weeks of age, in our hands (Cvetanovic, 2015; Qu et al., 2017; Ferro et al., 2018). Neuroprotective factors may compensate for early mutant ATXN1-mediated neural toxicity, and gradual decline in compensation may contribute to late onset of disease. Indeed, pre-manifest carriers of SCA1 and other polyQ mutations demonstrate mild coordination deficits and brain abnormalities several years prior to estimated disease onset (Jacobi et al., 2013; Storey, 2013; Tabrizi et al., 2013; Espinoza et al., 2018; Joers et al., 2018). We and others have observed cellular and molecular alterations that may compensate for neuronal dysfunction during the early stages of disease in *ATXN1[82Q]* mice (Dell’Orco et al., 2015; Kim et al., 2018). Here, we propose that increased expression of brain derived neurotrophic factor (BDNF) by reactive astroglia may be a part of this compensatory mechanism to delay disease onset in *ATXN1[82Q]* mice.

## Materials and Methods

### Human RNAseq

Reads per kilobase million (RPKM) RNAseq data was obtained from the BrainSpan database^1^ with permission (Miller et al., 2014). RPKM values were averaged over patients with equal ages and then normalized as follows:

$$Relative\;Expression=(RPKM_{n}-RPKM_{y})/(RPKM_{o}-RPKM_{n});$$

y, youngest datapoint; o, oldest datapoint.

### Mice

The creation of the *ATXN1[82Q]* mice was previously described (Clark et al., 1997). Equal number of male and female mice were randomly allocated to BDNF (20 μg of human recombinant BDNF (R&D Systems Cat. 248-BD-250/CF) in 100 μl per micropump, ~ 0.71μg/day) or control artificial cerebrospinal fluid (aCSF) groups. We surgically implanted ALZET pumps (Alzet Model 1004) into 8-week-old mice in a subcutaneous pocket in the back and the delivery cannula in the right lateral ventricle (A/P, 1.1; M/L, 0.5 D/V, −2.5 mm from Bregma) as we previously described (Cvetanovic et al., 2011).

In all the experiments investigators were blinded to the genotype/treatment.

Animal experimentation was approved by the Institutional Animal Care and Use Committee (IACUC) at the University of Minnesota and was conducted in accordance with the National Institutes of Health’s (NIH) Principles of Laboratory Animal Care (86-23, revised 1985), and the American Physiological Society’s Guiding Principles in the Use of Animals.

### Mouse RNA Sequencing

Mouse RNA sequencing was done as previously described (Ingram et al., 2016).

### Rotarod Analysis

Mice were tested on rotatod (#47600; Ugo Basile) to evaluate motor deficits as described previously (Kim et al., 2018). Rotarod paradigm consisted of acceleration from 5 rpm to 40 rpm over 0–5 min, followed by 40 rpm constant speed from 5 min to 10 min. Latency to fall was recorded.

### Immunofluorescent (IF) Staining

Immunofluorescent (IF) was performed on floating 45 μm brain sections using primary antibodies [Calbindin #C9848, Sigma-Aldrich; vesicular glutamate transporter 2 (VGLUT2) #MAB5504, Millipore, Burlington, MA, USA] as previously described (Cvetanovic et al., 2011).

Quantitative analysis was performed using ImageJ National Institutes of Health’s (NIH) as described previously. We quantified minimum of six different slices from each mouse. Per each slice we randomly chose two lobules and draw a line from the base of the Purkinje soma to the end of the dendrites to determine width of the molecular layer (length of the line) and average calbindin intensity. For assessing climbing fiber height, the distance from the Purkinje neuron soma to the end of VGLUT2 staining was measured (VGLUT2), and the extension of climbing fibers was depicted relative to the molecular layer thickness (VGLUT2/calbindin; Joers et al., 2018).

### Reverse Transcription and Quantitative Polymerase Chain Reaction (RT-qPCR)

Total RNA was extracted from mouse cerebella using TRIzol (Life Technologies) and (reverse transcription and quantitative polymerase chain reaction (RT-qPCR) was performed as described previously (Kim et al., 2018).

### Statistical Analysis

Wherever possible, sample sizes were calculated using power analyses based on the standard deviations from our previous studies, significance level of 5%, and power of 90%. Statistical tests were performed with GraphPad Prism. We have used one-way analyses of variance (ANOVA) followed by Bonferroni or Kruskal-Wallis *post hoc* tests (depending on the normality of the data) or two-tailed Student’s *t*-test.

### Data Availability

All the data from this study are available from the authors.

## Results

### BDNF Expression Is Altered in *ATXN1[82Q]* Mice

We used unbiased RNA sequencing to examine gene expression changes during disease progression. We have found that expression of neuroprotective factor BDNF is increased early and decreased late in *ATXN1[82Q]* mice (Cvetanovic et al., 2015; Supplementary Table S1), 5-week-old *ATXN1[82Q]* mice 6.87 vs. 5.3 fragments per kilobase of exon per million reads mapped (FPKM) in wild-type (WT) littermates, *p* = 0.00005, adjusted *q* = 0.0165; 12-week-old *ATXN1[82Q]* mice 3.2 vs. 5.03 fragments per kilobase of exon per million reads mapped (FPKM) in WT littermates, *p* = 0.00015, adjusted *q* = 0.0021). Using RT-qPCR we have confirmed increased BDNF expression in the cerebella during early stage (Figure 1C, 1.934 ± 0.359 relative to WT littermates, *N* = 5 each, Student’s *t*-test *P* = 0.0394) and later decrease (Figure 1D, 0.56 ± 006 relative to WT, *N* = 6 *ATXN1[82Q]* and *N* = 5 WT mice, Student’s *t*-test *P* = 0.0014).

Hallmark features of astrogliosis, the process by which astroglia react to neuronal dysfunction, are detectable early in SCA1 mice (Cvetanovic et al., 2015). We demonstrated that reducing astrogliosis early in SCA1, through inducible genetic inhibition of nuclear factor κ-light-chain-enhancer of activated B cells (NF-κB) signaling selectively in astroglia (astroglial IKKβ knock-out, aIKKβ KO), exacerbates motor deficits and neuropathology in *ATXN1[82Q]* mice (Kim et al., 2018). Inhibition of astroglial NF-κB prevented the early increase in BDNF expression (Figure 1E, 2.57 ± 0.11 increase in *ATXN1[82Q]* mice compared to 0.88 ± 0.17 in *ATXN1[82Q];aIKKB KO*, *N* = 4, *t*-test *P* = 0.024), indicating that astroglial NF-κB signaling may regulate increased BDNF expression early in SCA1 mice.

### Delivery of BDNF Early in Disease Ameliorates SCA1

**Figure 2:**
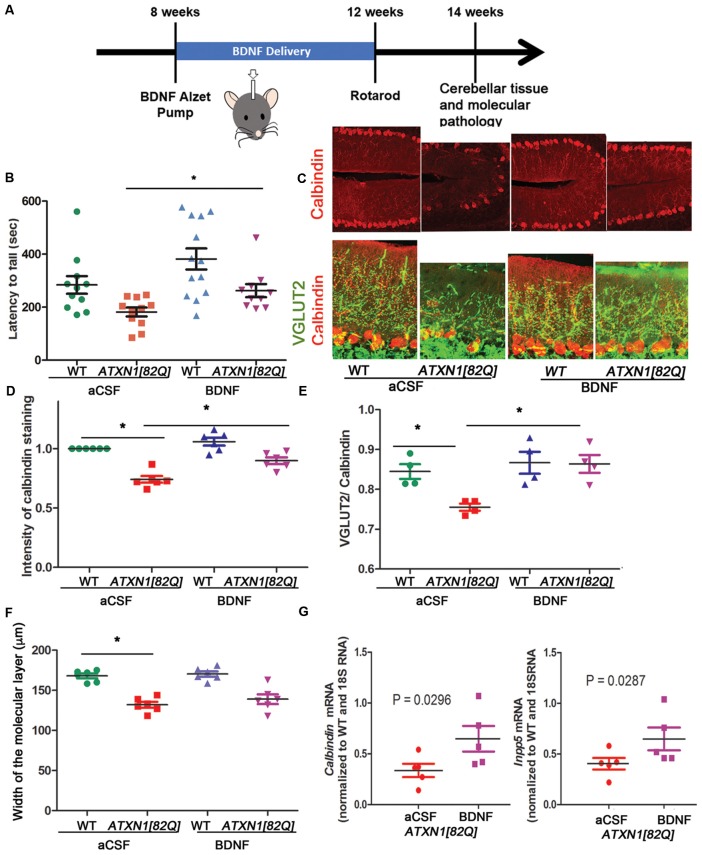
BDNF delivery delays disease pathogenesis in *ATXN1[82Q]* mice. **(A)** Experimental scheme to test the role of BDNF early in SCA1.** (B)** Rotarod performance of mice at 12 weeks. *P < 0.05 using one-way ANOVA with Kruskal-Wallis test. **(C–F)** Cerebellar slices from 14-week-old mice were stained with antibody specific for Purkinje neuron-marker calbindin and vesicular glutamate transporter 2 (VGLUT2) to label climbing fiber synapses on Purkinje neurons. ImageJ was used to quantify **(D)** calbindin intensity in the Purkinje neurons, **(E)** length of climbing fiber synapses (VGLUT2 puncta) on Purkinje neuron dendrites (determined as VGLUT2/calbindin ratio), and **(F)** width of the molecular layer. *P < 0.05 using one-way ANOVA with Bonferroni’s multiple comparison test. **(G)** RT-qPCR analysis of disease associated Purkinje neurons genes *calbindin* (left) and* Inpp5* (right). Student’s *t*-test *P* values. For all error bars = SEM. Each dot represents a biological sample.

In our hands, *ATXN1[82Q]* mice demonstrate reproducible motor deficits and cerebellar pathology earliest at 12 weeks. However, as the onset of motor deficits has been observed earlier by others (Clark et al., 1997; Duvick et al., 2010; Hourez et al., 2011; Ibrahim et al., 2017), we refer to mice younger than 12 weeks as early stage instead of pre-symptomatic. To determine the neuroprotective effects of BDNF, we used osmotic ALZET pumps to deliver recombinant BDNF to 8-weeks-old SCA1 or WT littermate mice for a total of 4 weeks (~0.71 μg/day). This study consisted of four experimental groups: WT and *ATXN1[82Q]* mice with pumps delivering BDNF, and control WT and *ATXN1[82Q]* mice with pumps delivering aCSF. Mice were tested on rotarod at 12 weeks and pathology was examined at 14 weeks (Figure 2A).

BDNF-treated *ATXN1[82Q]* mice performed significantly better on rotarod compared to control aCSF-treated *ATXN1[82Q]* mice (e.g., day 4 average latency to fall of BDNF-treated *ATXN1[82Q]* mice = 262.5 ± 24.3 s, *N* = 10; compared to aCSF-treated *ATXN1[82Q]* mice = 181 ± 16.73 s, *N* = 11, *P* < 0.05, one-way ANOVA with Kruskal-Wallis test *H* = 15.74, *P* = 0.0013; Figure 2B). Importantly, BDNF-treated *ATXN1[82Q]* mice were indistinguishable from BDNF- or aCSF-treated WT mice (aCSF- and BDNF- treated WT mice latency was 283 ± 36.7 s, *N* = 12, and 382 ± 39.78, *N* = 13, respectively, *P* > 0.05 for both comparisons one-way ANOVA with Kruskal-Wallis test).

We used immunofluorescence to determine whether BDNF ameliorates decreased calbindin expression and synaptic loss, two well-characterized hallmarks of Purkinje neuron pathology in SCA1 mice (Zu et al., 2004; Serra et al., 2006; Duvick et al., 2010; Barnes et al., 2011; Ebner et al., 2013; Ruegsegger et al., 2016; Ibrahim et al., 2017). BDNF-treated *ATXN1[82Q]* and WT mice were indistinguishable and both groups had significantly higher calbindin intensity of Purkinje neurons compared to aCSF-treated *ATXN1[82Q]* controls (Figures 2C,D, average relative intensity of aCSF-treated *ATXN1[82Q]* mice was 0.7417 ± 0.028 compared to 0.8989 ± 0.027 in BDNF-treated *ATXN1[82Q]* mice, *N* = 6 of each, *P* < 0.05 one-way ANOVA with *post hoc* Bonferroni test *F*_(3,20)_ = 29.32, *P* < 0.0001). BDNF also prevented loss of climbing fibers synapses on Purkinje neuron dendrites in *ATXN1[82Q]* mice as quantified with VGLUT2 staining (Figures 2C,E, average VGLUT2/CAL ratio for aCSF-treated *ATXN1[82Q]* mice was 0.7553 ± 0.009 compared to 0.8638 ± 0.02 in BDNF–treated *ATXN1[82Q]* mice, *N* = 4 each, one-way ANOVA *F*_(3,12)_ = 3.878 *P* = 0.0071, *post hoc* Bonferroni test *P* < 0.05). However, width of the molecular layer was not rescued (Figure 2F, average width for aCSF-treated *ATXN1[82Q]* mice was 132.1 ± 3.667 μm compared to 139 ± 6.1 in BDNF–treated *ATXN1[82Q]* mice, *N* = 6 each, one-way ANOVA with *post hoc* Bonferroni test *P* > 0.05).

We used RT-qPCR to demonstrate that BDNF treatment rescued expression of Purkinje neuron genes *Calbindin* (Ingram et al., 2016; Figure 2G left, aCSF-treated *ATXN1[82Q]* mice = 0.336 ± 0.065 compared to 0.648 ± 0.125 in BDNF-treated *ATXN1[82Q]* mice, *N* = 5, *t*-test, *P* = 0.0296) and *INNP5* (Figure 2G right, aCSF-treated *ATXN1[82Q]* mice = 0.404 ± 0.057 compared to 0.648 ± 0.115 in BDNF-treated *ATXN1[82Q]* mice, *t*-test, *P* = 0.02872, *N* = 5). Together, these results indicate that BDNF may be neuroprotective early in SCA1.

## Discussion

We report that early delivery of BDNF delays motor deficits and pathology of Purkinje neurons. In addition, we report an early increase in BDNF expression in SCA1 that may be regulated by astroglial NF-κB-signaling. Based on these results, we propose that early increase in BDNF expression may be neuroprotective and compensate for ATXN1 toxicity, thus delaying disease onset.

BDNF belongs to the family of neurotrophins (Barde et al., 1982) and is expressed in human cerebellum from early development (Figure 1B). Its cognate receptor, Tyrosine kinase B receptor (TrkB), is highly expressed on Purkinje neurons where BDNF signaling plays a host of neuroprotective and modulatory functions such as regulating dendritic branching and synaptic strength (Schwartz et al., 1997; Kafitz et al., 1999; Carter et al., 2002; Furutani et al., 2006; Huang et al., 2012). Purkinje neuron pathology in SCA1 includes shrinking of the dendritic arbor, loss of synapses, and altered evoked and spontaneous firing (Duvick et al., 2010; Cvetanovic et al., 2011; Hourez et al., 2011; Dell’Orco et al., 2015). It is possible that BDNF exerts its neuroprotective effect in SCA1 at least in part through prevention of these Purkinje neuron pathologies. Intriguingly, during postnatal cerebellar development BDNF signaling seems to promote elimination of climbing fiber synapses, and this may be regulated by temporal expression of short TrkB isoform (Bosman et al., 2006; Sherrard et al., 2009; Choo et al., 2017). Delivery of BDNF to WT mice at 8 weeks did not seem to affect climbing fiber synapses and these mice performed well on rotarod (Figure 2B) indicating that post-development extrinsic BDNF may not have obvious side effects on these tests. Since BDNF is delivered intracerebroventricularly, we cannot exclude the possibility of off-target BDNF effects. However, *ATXN1[82Q]* mice pathology is limited to cerebellum (Burright et al., 1995) due to Purkinje neuron selective expression of mutant ATXN1.

BDNF expression is decreased in many neuropsychiatric and neurodegenerative diseases, including depression, bipolar disease, schizophrenia, Alzheimer’s disease, HD, SCA6 and Parkinson’s disease (Ferrer et al., 2000; Zuccato and Cattaneo, 2007; Takahashi et al., 2012; He et al., 2013; Shin et al., 2015). Moreover, beneficial treatments for these diseases correlate with increased BDNF expression, and rescued BDNF expression ameliorates disease symptoms in mouse models (Willson et al., 2008; Nagahara et al., 2009; Giampà et al., 2013). While these studies underlie the importance of BDNF in brain pathology and pathophysiology, regulation of BDNF expression during disease is less understood. Recent studies indicate a link between BDNF and neuroinflammation (Lima Giacobbo et al., 2018), another phenomenon that is ubiquitously present in these diseases (Ilieva et al., 2009; Heneka et al., 2014; Pekny et al., 2016). NF-κB, a key regulator of neuroinflammation (Mincheva-Tasheva and Soler, 2013), modulates BDNF expression (Saha et al., 2006; Lima Giacobbo et al., 2018). Astroglia react to neuronal dysfunction by activating NF-κB (Barres, 2008), and our results implicate that astroglial NF-κB contributes to elevated BDNF levels early in SCA1 mice. Likewise, astroglia-derived BDNF delayed disease onset in a transgenic mouse model of HD (Giralt et al., 2011).

Previous studies indicate that reversibility of polyQ neurodegeneration diminishes with disease progression (Yamamoto and Lucas, 2000; Zu et al., 2004; Rubinsztein and Orr, 2016; Ibrahim et al., 2017), strongly supporting consideration of early stage treatments. Our results indicate that early BDNF treatment may delay disease onset in SCA1 carriers. We also observed decreased BDNF expression late in SCA1 that may contribute to disease severity (Hourez et al., 2011). Future work will examine the effects of late BDNF delivery on disease severity and progression in SCA1 mice.

## Author Contributions

MC conceived the study. AM, MC and CS performed the experiments and analyzed the data. AF analyzed data from BrainSpan database linked from Allen brain atlas. All authors prepared the figures and wrote the manuscript.

## Conflict of Interest Statement

The authors declare that the research was conducted in the absence of any commercial or financial relationships that could be construed as a potential conflict of interest.

## Footnotes

^1^http://www.brainspan.org/

## Supplementary Material

TABLE S1 BDNF expression from RNA sequencing of mouse cerebellar extracts.
