# Supplementary figures and images for: Brain Derived Neurotrophic Factor (BDNF) Delays Onset of Pathogenesis in Transgenic Mouse Model of Spinocerebellar Ataxia Type 1 (SCA1)
Source: Front Cell Neurosci. 2019 Jan 21;12:509. doi: 10.3389/fncel.2018.00509 (PMC6348256; doi:10.3389/fncel.2018.00509)

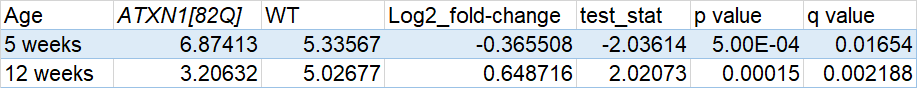

Supplement: TABLE S1 — BDNF expression from RNA sequencing of mouse cerebellar extracts. [file Image_1.TIF]
